# LONG-TERM FOLLOW-UP OF FUNCTIONING AND LIFE SATISFACTION AFTER MODERATE TO SEVERE TBI – AN EXPLORATORY COHORT STUDY

**DOI:** 10.2340/jrm-cc.v8.44100

**Published:** 2025-12-18

**Authors:** Marianne LANNSJÖ, Jörgen BORG, Charlotta VON SETH, Per ENBLAD, Sami ABU HAMDEH

**Affiliations:** 1 Section of Rehabilitation Medicine, Department of Medical Sciences, Uppsala University, Uppsala, Sweden; 2 Division Rehabilitation Medicine, Department of Clinical Sciences, Danderyd Hospital, Karolinska Institutet, Stockholm, Sweden; 3 Department of Medical Sciences, Uppsala University, Uppsala, Sweden; 4 Section of Neurosurgery, Department of Medical Sciences, Uppsala University, Uppsala, Sweden

**Keywords:** traumatic brain injury, diffuse axonal injury, long-term outcome, life satisfaction

## Abstract

**Objective:**

To examine the long-term outcomes after moderate to severe traumatic brain injury with diffuse axonal injury.

**Design:**

A follow-up study of a regional cohort with a mean follow-up period of 5 years post-injury.

**Subjects:**

Thirty-four participants were recruited from a regional neurointensive care unit. Inclusion criteria were age 16–65 years, a Glasgow Coma Scale score of 3–12, and radiologically confirmed diffuse axonal injury.

**Methods:**

Follow-up assessments conducted at least 1 year post-injury included global outcome measured by the Glasgow Outcome Scale Extended, activities of daily living assessed with the Barthel Index, and life satisfaction evaluated using the LiSat checklist.

**Results:**

More than two-thirds of participants regained independence, and approximately half returned to work or studies. A higher diffuse axonal injury stage was associated with a lower likelihood of returning to work or studies. Over half of respondents reported being satisfied or very satisfied with “life as a whole.” Return to work or study was significantly associated with life satisfaction, and there was a trend toward higher satisfaction among those living with a partner.

**Conclusion:**

These findings confirm and extend observations from previous studies, emphasizing key areas for rehabilitation and long-term outcome after moderate to severe traumatic brain injury with diffuse axonal injury.

## LAY ABSTRACT

Knowledge about the long-term outcomes following the most severe traumatic brain injuries with diffuse axonal injury remains limited, particularly regarding life satisfaction, degree of independence, leisure activities, and close relationships. In this study, 34 participants aged 16–65 years from a regional neurointensive care unit were followed up at a mean of 5 years post-injury. Findings showed that more than two-thirds had regained independence, approximately half had returned to work or studies, and about half of those able to respond reported being satisfied with life as a whole. Return to work or study was associated with higher life satisfaction, and living with a partner showed a trend toward greater satisfaction.

Traumatic brain injury (TBI) remains a concern globally (1). One recent review reported the incidence of TBI in Europe to be 118 to 546 per 100,000 per year (2) when all severity grades were included and 4.1 to 17 per 100,000 per year when only severe injuries were included.

Even though group-level outcome after TBI is related to the initial injury severity, individual patients may still have a good outcome after a severe TBI. In the Track-TBI study (3), outcome at 12 months after TBI was favourable (defined as a Glasgow Outcome Scale Extended [GOSE] score of 4–8) in 52% of those with severe TBI and in 75% of those with moderate TBI. Diffuse axonal injury (DAI) is associated with poor outcome after severe TBI (4). However, several studies indicate that a substantial proportion of patients with DAI may have a favourable outcome at 1 year after the injury (5, 6) even though findings differ based on the definition of favourable outcome and the mix of injury severity and location. Injury in the brainstem has been found to be related to unfavourable outcome (6, 7). The severity of DAI is commonly classified according to the system developed by Adams and colleagues in three stages (1. microscopic-only evidence of axonal damage in cerebral hemispheres, 2. addition of focal lesion in corpus callosum, 3. addition of focal lesion in the brain stem). In one study, the odds ratio (OR) for unfavourable outcome (defined as GOSE 1–5) increased three times for each grade of DAI (8). The Adams classification system is, however, developed from postmortem studies and does not account for lesion volume, bilaterality or injuries in specific brainstem structures. Therefore, new classification systems based on conventional magnetic resonance imaging (MRI) have been proposed during the last decade to improve prognostic accuracy. Abu Hamdeh et al introduced an extended anatomical grading, adding a fourth stage with lesions in the substantia nigra and mesencephalic tegmentum to the original Adams classification (9). Similarly, the Stockholm MRI grading system and the Trondheim traumatic axonal injury-magnetic resonance imaging (TAI-MRI) grading system both emphasize the association between bilateral brainstem lesions and poor outcomes (10, 11).

Reports on outcome more than 1 year after TBI with DAI are scarce and findings are not consistent. In one study with follow-up at 3–6 years after the injury (mean 5 years), DAI did not predict GOSE (12). Another study (13) with a follow-up after a median of 54 months (range 14–100) reported favourable outcome (GOSE 6–8) in 39% of patients with DAI III and in 47% of patients with DAI II. Recently, we reported that DAI stage together with age were significant predictors of long-term outcome (mean 5 years, range 1–14 years) (14).

GOSE is an internationally established instrument for assessing outcome after TBI. GOSE captures aspects of the ability to work and being independent, but only partly captures leisure-time activities or relations with spouse, children and others. These perspectives may also be important for the individual and may impact on self-reported life satisfaction, as indicated in previous studies. Factors related to higher life satisfaction include employment (15–17), community participation (15, 17, 18), engagement in leisure activities (17) and lower degree of romantic loneliness (19). Overall, there are few studies on the outcome after TBI with DAI regarding life satisfaction, leisure-time activities and relationships with family and others (20). The aim of this study was to adress this knowledge gap by reporting long-term outcomes after moderate and severe TBI with DAI in a regional cohort followed for a mean of 5 years after the injury, with a focus on life satisfaction, return to work, marital status and leisure-time activities.

## MATERIALS AND METHOD

The Regional Research Ethics Committee granted permission for all included studies. Written informed consent was first obtained from the TBI patients’ closest relatives and from the patients themselves when they had sufficiently recovered from their injury at > 6 months post-injury. All research was conducted in accordance with the ethical standards as outlined in the 1975 Helsinki Declaration and its later amendments in 2008.

Participants included TBI patients treated at the neurointensive care (NIC) unit at Uppsala University Hospital between 2006 and 2018. Inclusion criteria were: 1) DAI diagnosis by MRI in the acute phase or by a combination of computed tomography (CT) and clinical assessment, 2) age 16–65 years and 3) moderate to severe injury according to Glasgow Coma Scale at admission (GCS 3–12). Exclusion criteria were: 1) earlier moderate-severe TBI, 2) neurological or psychiatric disease, 3) substance abuse and 4) other coincidental injury of major importance including diagnoses such as spinal cord injury, hypoxic disorder, major haemorrhage or other substantial injury that could have interfered with the treatment and recovery from the actual TBI. The initial brain injury was first analysed by CT at admission. Suspected DAI on the initial CT was subsequently confirmed with MRI when the patient was medically stable and possible to transfer to the radiology department.

All study participants were subject to early assessments by specialists in neurorehabilitation, who also designed individually adapted rehabilitation programmes.

### Radiological classification, patient data collection and follow-up evaluation

The brain injury was first analysed by CT. The stage of DAI was defined by Adams (21) and an extended anatomical grading suggested by Abu Hamdeh et al (9), where stage IV was defined as haemorrhagic or non-haemorrhagic lesions in the substantia nigra and mesencephalic tegmentum. Data regarding GCS and cause of injury were collected from the Uppsala TBI register (22).

A follow-up interview 1 year or later after injury was made by telephone or by a visit. It consisted of a comprehensive assessment which included estimation of GOSE, length of post-traumatic amnesia (PTA), activities of daily living (ADL) functioning by Barthel, life satisfaction – item “life as a whole” – by LiSat.

### Glasgow Outcome Scale Extended

GOSE is a widely used instrument for assessing global outcome after TBI and has been shown to be valid and reliable (23). It consists of an ordinal scale with 8 steps where 1 = dead, 2 = vegetative state, 3 = lower severe disability, 4 = upper severe disability, 5 = lower moderate disability, 6 = upper moderate disability, 7 = lower good recovery, 8 = upper good recovery. Favourable outcome was defined as GOSE 6–8. The structured interview designed to improve the assessment of GOSE (24) was used to assess GOSE at the follow-up interview.

### Barthel Index

The Barthel Index for ADL is an ordinal scale which measures a person’s ability to complete ADL. The 10 items are scored with 0, 5 or 10 points and the total score then varies between 0 (which means total independence) and 100 (which means that the person is totally dependent) (25).

### Rivermead Posttraumatic Amnesia Protocol

The Rivermead Post-Traumatic Amnesia protocol estimates the length of post-traumatic amnesia (PTA) through clinical questioning of the patient. Four grades of PTA are defined by establishing how long after injury (in hours/days/weeks) the patient regains continuous day to day memory: mild = less than 1 h, moderate = 1–24 h, severe = 1–7 days, very severe = more than 7 days (26).

### Life satisfaction checklist LiSat-11

This is a commonly used instrument in Sweden, included in the Swedish National Quality-Registry for Rehabilitation Medicine (27). The questionnaire consists of 11 questions where the participant indicates the level of satisfaction. The scale is ordinal with six levels from “Very dissatisfied” to “Very satisfied.” LiSat-11 has been found to be valid for the general population (28). The answers can be dichotomized into two groups, satisfied (“satisfied, very satisfied”) and dissatisfied (“very dissatisfied, dissatisfied, rather dissatisfied, rather satisfied”). Validity has been proven both for the whole instrument and for each item separately. In this study, only the global aspect “life as a whole,” which represents a global measure of satisfaction with life (28), was used.

### Statistics

All statistical analyses were conducted in SPSS version 27 (IBM Corp., Armonk, New York, USA). Χ^2^ and Mann Whitney U tests were performed to test statistical differences between groups. Multiple Χ^2^ tests were performed for multiple group comparison, followed by pairwise post hoc tests if significant differences were found. A *p*-value < 0.05 was considered statistically significant.

## RESULTS

Patient characteristics are presented in Table I. There were 67 patients who were eligible for the study, treated during the years 2006–2018. Eight of them died before follow-up and 25 either declined participation or did not answer despite 2 or 3 reminders. Thus, the study included 34 participants (24 men, 10 women), aged 16–61 years (mean 33). Based on GCS, there were 31 participants with severe TBI (GCS 3–8) and 3 with moderate TBI (GCS 9–12). Twenty-two of the 34 participants had DAI stage III based on Adams’ classification and 16 of them had DAI stage IV, based on Abu Hamdeh’s classification.

**Table I:** Demografic and injury-related data

Variable		No
Sex	Men	24
	Women	10
GCS	3–8 Severe injury	31
	9–12 Moderate injury	3
DAI stage according to Adams*	I	3
	II	7
	III	22
DAI stage according to Abu Hamdeh*	I	3
	II	7
	III	6
	IV	16
Cause of injury	Traffic accident, car	20
	Traffic accident, bicycle	3
	Traffic accident, pedestrian	4
	Sports accident	5
	Fall	1
	Hit by object	1

* Missing data regarding grade of DAI for 2 participants.

GCS: Glasgow Coma Scale; DAI: diffuse axonal injury.

Follow-up was performed after 1–14 years (mean 5). Twenty-three participants were followed-up by a visit and 11 by a telephonic interview.

Non-responders (*n* = 24) did not differ in age (mean 30, *p* = 0.40), sex (20 men, 4 women, *p* = 0.26) or GCS (21 with GCS 3–8, 3 with GCS 9–12, *p* = 0.48).

### PTA and Glasgow outcome score extended

Twenty-eight participants had a PTA longer than 7 days, indicating severe amnesia. Data were missing for six participants, of which four were classified as GOSE 3 and were unable to contribute in estimating PTA. It was estimated that PTA was longer than 7 days in those cases. Thus 32 participants had a PTA longer than 7 days, with data missing for two. GOSE outcomes showed 22 participants with unfavourable (GOSE 3–5) and 12 with favourable outcome (GOSE 6–8).

### Outcome regarding working or studying ability

At the follow-up, 11 of the participants were working or studying full-time, three were working or studying part-time and three were in work-training. The remaining 17 participants were neither working nor studying. Prior to the injury, most of the participants (*n* = 20) were working and eight participants were students (Table IV).

A significant relation was found between DAI-stage and ability to work or study at follow-up (Table II). Individuals with DAI stage IV had a worse work/study outcome compared to those with DAI stage I (p=0.013) and DAI stage II (p=0.035), and showed a trend toward significance compared to DAI stage III (p=0.07).

**Table II:** Diffuse axonal injury (DAI) grade according to Abu Hamdeh and work/study situation at follow-up

DAI	Work/study at follow-up
I (3)	3
II (7)	5
III (6)	4
IV (16)	4
Not graded (2)*	1

* There were 2 participants who did not undergo an magnetic resonance imaging but they were diagnosed as DAI according to computed tomography and clinical assessment.

### Change in situation regarding work/studies, leisure time and living situation after the traumatic brain injury

At follow-up, the participants were asked, according to a structured interview, about changes in work or study status, leisure activities, and living situation after the injury (Table III). A total of 25 reported a decline in their ability to work or study. Of these, eight participants were still working or studying, including two full-time, but reported reduced capacity. Overall, the ability to work was more affected than leisure time activities and living situation compared with the period before the TBI.

**Table III:** Participants’ subjective opinion of life situation at follow-up compared to before TBI

Variable	Unaltered	Improved	Impaired
Work/studying	6	3	25
Leisure time*	15	2	16
Living situation*	20	5	8

* Missing data for 1 participant. TBI = traumatic brain injury.

### Outcome regarding activites in daily life, living situation and marital status

Functioning in personal ADL was assessed using Barthel index and interview data (Table IV). Twenty-six of the participants were independent in ADL, three were nearly independent and five were highly or completely dependent. Nineteen of the participants were self-sufficient in their living situation, six had some support and nine had extensive support.

**Table IV:** Status at follow-up regarding living situation, ability in ADL, marital status and working/studying situation

Variable	Status		At follow-up
ADL	Independent (34)		26
	Some or total support (0)		8
Living situation	Independent (34)		19
	Some or total support (0)		15
Marital status	Unchanged		20
	From single (17) to living with a partner		5
	From living with a partner (17) to single		9
Working / Studying	Work (25)		10*
	Designated work (1)		0
	Studying (8)	to studying	4
		to work	3**

Situation before injury within brackets.

* Two of the participants were in work-training or had a designated work.

** One of the participants had a designated work. ADL: activities in daily life.

Twenty of the participants, equally spread among GOSE 3–8, reported no change in their living situation; eight continued to live with a partner and 12 remained single. Following the injury, nine participants had changed marital status to living alone, all of them classified as GOSE 3–5. Five of the participants had changed marital status to living with a partner, four of which were classified as GOSE 6–8.

### Life satisfaction according to the LiSat Checklist

Only the first variable in LiSat was collected – life as a whole (Table V). There were missing data for 11 participants, eight of which were classified as GOSE 3 or 4 and were not capable of reporting the level of satisfaction. The ability to work or study was significantly associated with life satisfaction (*p* = 0.008). Marital status showed a trend towards higher satisfaction among those living with a partner (*p* = 0.06). ADL-function (*p* = 0.09) and support in living situation, such as assistance with cleaning and laundry, did not affect the level of life satisfaction (*p* = 0.35).

**Table V:** Life satisfaction related to work/studying, marital status, ADL ability and support in living situation

Variable	LiSat 3–4 (no 10)	LiSat 5–6 (no 13)	LiSat missing (no 11)
Working or studying (17)	3 (30%)	11 (85%)	3 (27%)
Not working or studying (17)	7 (70%)	2 (15%)	8 (73%)
Living with partner (12)	1 (10%)	7 (54%)	4 (36%)
Living as single (22)	9 (90%)*	6 (46%)*	7 (64%)
ADL independency (26)	8 (80%)	13 (100%)	5 (45%)
ADL dependency (8)	2 (20%)	0	6 (55%)
Self-sufficient in living situation (19)	5 (50%)	9 (69%)	5 (45%)
Support (not ADL) in living situation (15)	5 (50%)	4 (31%)	6 (55%)

No participant rated life satisfaction to LiSat 1 or 2.

* One participant lived with parents. ADL = activities in daily life.

## DISCUSSION

All participants in this study had suffered a moderate or severe TBI according to GCS. However, all patients experienced a PTA duration longer than 7 days and can, according to PTA outcomes, be classified as having sustained a severe TBI. The vast majority exhibited severe DAI ≥ III. The main findings were that at long-term follow-up, more than two-thirds had regained self-independence, around half had returned to work and more than half of those who were able to respond were satisfied or very satisfied with “life as a whole.” A third of the participants had a favourable outcome according to GOSE. The choice of GOSE 6–8 as favourable outcome was on the basis that the individual could work or study to some extent, be independent, perform activities at least to some degree and participate meaningfully in society.

Good or enough satisfaction with life may be considered the essence of good outcome after TBI. In this study, life satisfaction was assessed by using the LiSat scale. Reference data from a national reference population (28) exist for this scale. In a study conducted by Jacobsson and Lexell (29), the item “Life as a whole” was found to be strongly correlated with the total score of Satisfaction With Life Scale (SWLS). That allows comparison with data from studies using SWLS. When dichotomizing LiSat, level 5–6 stands for satisfied and level 1–4 for not satisfied (28). More than a half of those who could respond, rated LiSat 5 or 6, which means satisfied or very satisfied with “life as a whole,” and no one rated 1 or 2 (meaning very dissatisfied or dissatisfied). The median LiSat score for the age-matched reference population was five, indicating that many study participants reported life satisfaction at a similar high level. Further, similar to the reference population, as well as findings in a 6 years follow up after stroke (30), factors related to “Closeness” such as partner relationship were associated with the reported satisfaction with “life as a whole” (28).

Previous TBI studies have also pointed out that relations to close relatives and especially to a partner are important for life satisfaction (19, 31). Other factors of importance for life satisfaction after TBI include being able to work or study, being independent in ADL, having good social relations, not living alone and being able to perform leisure activities (15, 17–19). The observations in the current study of severely injured persons are in line with those findings. The ability to work or study was significantly associated with life satisfaction (*p* = 0.008), and marital status showed a trend towards higher satisfaction among those living with a partner. Being independent in ADL was reported by more than two-thirds (24 of 34) of the participants, and more than a half were self-sufficient in their living situation. The Swedish Insurance System provides funding for personal assistants who can support individuals in their homes with tasks such as feeding, cleaning, laundry, transports and other daily activities. Therefore, individuals are not required to live in a nursing home. Despite this, it can be very valuable for the individual to be able to manage their living situation independently. The proportion of ADL-independent participants was higher than the proportion that returned to work or studies, which may reflect persisting cognitive disability. It should be noted that DAI-stage was significantly related to outcome regarding work or studying. The higher the DAI-stage, the lower the degree of return to work or studies. How these factors interact among each other deserves further studies in order to optimize personalized rehabilitation plans.

When comparing the participants’ subjective opinion of change in life situation after TBI, impairment in the working or studying situation was the most frequently reported change. This was not unexpected, although 6 of the 17 participants were working or studying, had a designated job, or were in work training, and thus had experienced a major change in their work or study situation. Also, even if working or studying at the same level as before the TBI, participants can experience an impaired capacity to work or study. Perceived impairments in living situation and leisure time activities were less common but may nonetheless be of importance for life satisfaction. Those who reported impairment in working or studying situation also reported impairment in leisure time activities and living situation. This demonstrates that abilities and activities are closely linked to each other and that ability to work or study seems to be the most vulnerable area.

In our earlier study (14), we found that most of the participants showed little change in GOSE between 6 months after injury and the late follow-up. Therefore, it was valuable to describe outcomes in additional aspects, such as life satisfaction and marital status. However, it should be noted that improvement may continue into the second and third year after a severe injury, which means that findings from participants followed up after 1 or 2 years may be less reliable than those from participants followed up later.

The findings in this study should reflect the results of modern prehospital management and NIC (32, 33) as well as access to specialised brain injury rehabilitation (34–36). The findings are in accordance with previous reports which indicate that going back to work or studies should be prioritised in the rehabilitation process (15, 18) and lend further support for the impact of functioning in relation to close relatives as highlighted by Prigatano already in 1989 (31). From a neurorehabilitation perspective, there is a need for more research on peer-support, continuity of care and equality in service delivery not least in the long-term perspective for young people (20).

### Limitations

One main limitation of this study is the small study sample, which limits statistical analyses and conclusions that can be drawn. However, the study sample did not differ from non-responders in age, sex, or GCS, and can therefore be considered representative. Data were incomplete for assessment of LiSat, mainly due to inability to respond for those with the most severe injuries, which is an inherent limitation in this group of patients. Outcome measured as early as 1 year after the injury could in some cases appear worse than the final outcome, both due to potential improvement later than 1 year and due to temporary obstacles such as leg fracture or other factors affecting the ability to work, study or move from the family home to an independent accommodation. However, regarding the scarcity of consistent data on long-term outcome after severe TBI with DAI, we believe our observations may be valuable for the management of these patients and the planning of further studies.

### Conclusions

In this exploratory cohort study of patients with moderate to severe TBI and DAI with long-term follow-up, more than half of responding participants were satisfied or very satisfied with life. There was a significant association between life satisfaction and ability to work or study, and the association to living with a partner was nearly significant. Ability to return to work or studying was significantly related to DAI-stage. The results lend strong support to rehabilitation efforts regarding return to work and to provide and develop strategies to support relations with close relatives.

*The authors have no conflict of interest to declare*.
